# Baricitinib inhibits structural joint damage progression in patients with rheumatoid arthritis—a comprehensive review

**DOI:** 10.1186/s13075-020-02379-6

**Published:** 2021-01-04

**Authors:** Paul Emery, Patrick Durez, Axel J. Hueber, Inmaculada de la Torre, Esbjörn Larsson, Thorsten Holzkämper, Yoshiya Tanaka

**Affiliations:** 1 Leeds Institute of Rheumatic and Musculoskeletal Medicine, University of Leeds, NIHR Leeds BiomedicalResearch Centre, Leeds Teaching Hospitals NHS Trust, Leeds, UK; 2 Pôle de Pathologies Rhumatismales Inflammatoires et Systémiques, Institut de Recherche Expérimentale et Clinique, Université Catholique de Louvain and Service de Rhumatologie, Cliniques Universitaires Saint-Luc, Brussels, Belgium; 3 Section Rheumatology, Sozialstiftung Bamberg, Bamberg, Germany; 4 Eli Lilly and Company, Indianapolis, IN USA; 5 University of Occupational and Environmental Health, Kitakyushu, Japan

**Keywords:** Rheumatoid arthritis, Baricitinib, Radiographic progression, Joint damage, Joint erosion

## Abstract

Baricitinib is an oral selective inhibitor of Janus kinase (JAK)1 and JAK2 that has proved effective and well tolerated in the treatment of rheumatoid arthritis (RA) in an extensive programme of clinical studies of patients with moderate-to-severe disease. In a phase 2b dose-ranging study of baricitinib in combination with traditional disease-modifying antirheumatic drugs (DMARDs) in RA patients, magnetic resonance imaging showed that baricitinib 2 mg or 4 mg once daily provided dose-dependent suppression of synovitis, osteitis, erosion and cartilage loss at weeks 12 and 24 versus placebo. These findings correlated with clinical outcomes and were confirmed in three phase 3 studies (RA-BEGIN, RA-BEAM and RA-BUILD) using X-rays to assess structural joint damage. In patients naïve to DMARDs (RA-BEGIN study), baricitinib 4 mg once daily as monotherapy or combined with methotrexate produced smaller mean changes in structural joint damage than methotrexate monotherapy at week 24. Differences versus methotrexate were statistically significant for combined therapy. In patients responding inadequately to methotrexate (RA-BEAM study), baricitinib 4 mg plus background methotrexate significantly inhibited structural joint damage at week 24 versus placebo, and the results were comparable to those observed with adalimumab plus background methotrexate. In patients responding inadequately to conventional synthetic DMARDs (csDMARDs; RA-BUILD study), baricitinib 4 mg again significantly inhibited radiographic progression compared with placebo at week 24. Benefits were also observed with baricitinib 2 mg once daily, but the effects of baricitinib 4 mg were more robust. The positive effects of baricitinib 4 mg on radiographic progression continued over 1 and 2 years in the long-term extension study RA-BEYOND, with similar effects to adalimumab and significantly greater effects than placebo. Findings from the phase 3 studies of patients with RA were supported by preclinical studies, which showed that baricitinib has an osteoprotective effect, increasing mineralisation in bone-forming cells. In conclusion, baricitinib 4 mg once daily inhibits radiographic joint damage progression in patients with moderate-to-severe RA who are naïve to DMARDs or respond inadequately to csDMARDs, including methotrexate, and the beneficial effects are similar to those observed with adalimumab.

**Supplementary information:**

**Supplementary information** accompanies this paper at 10.1186/s13075-020-02379-6.

## Background

Rheumatoid arthritis (RA) is a chronic, inflammatory, autoimmune disease associated with structural joint damage leading to disability [1, 2]. Joint damage is caused by the destruction of cartilage and bone via the activation of chondrocytes and fibroblasts, leading to the production of metalloproteinases and osteoclasts (bone-resorbing cells) [2]. These events are driven by the overproduction of pro-inflammatory cytokines, such as tumour necrosis factor-α, interleukins-6 and -17 and macrophage colony stimulating factor, by immune cells in the synovium [3]. The prevention of damage to cartilage and bone is an important goal in the treatment of RA [4, 5], and agents that inhibit cytokine intracellular transduction pathways have therefore been investigated as possible treatments for the disease. One such pathway is the Janus kinase (JAK)/signal transducers and activators of transcription (STAT) pathway [6].

Baricitinib is an orally available small molecule that reversibly inhibits JAK1 and JAK2, thereby blocking cytokine signalling through the JAK/STAT pathway [6, 7]. The efficacy and safety of baricitinib as a treatment for RA have been confirmed in an extensive programme of clinical studies of patients with moderate-to-severe disease [8]. The results of these studies have shown that in addition to reducing disease activity, baricitinib inhibits radiographic progression of structural joint damage [9–12], provides effective pain relief [13, 14] and improves various patient-reported outcomes, including physical function, fatigue, work productivity and quality of life [13, 15–18]. Baricitinib is currently approved for the treatment of moderate-to-severe RA in adults in more than 70 countries worldwide, and more than 100,000 patients with RA have been treated with the drug to date (Eli Lilly & Company, data on file).

The aim of this review is to collate and summarise all data on the effects of baricitinib on structural joint damage progression and the mechanisms underlying these effects. Results achieved with approved doses of baricitinib (2 mg or 4 mg once daily, apart from in the USA, Canada and China, where the approved dose is 2 mg once daily), measured through magnetic resonance imaging (MRI) or radiographic progression of joint erosion and joint space narrowing, in clinical studies and post hoc analyses of patients with RA who are naïve to disease-modifying antirheumatic drugs (DMARDs) or have an inadequate response to conventional synthetic DMARDs (csDMARDs) are presented. In addition, data from preclinical studies of baricitinib are reviewed.

## MRI findings from a phase 2 study

The effects of baricitinib on joint damage progression were investigated in a phase 2 study (NCT01185353) in which adult patients with moderate-to-severe active RA despite treatment with methotrexate were randomised to placebo or once-daily baricitinib (1, 2, 4 or 8 mg) for 24 weeks [19]. Patients with radiographic evidence of joint erosion in the hands/wrist and feet underwent MRI of the hands/wrist at baseline and at weeks 12 and 24. The images were scored by two expert radiologists who were blinded to the chronologic order of the radiographs and treatment. Images were scored for synovitis, osteitis and bone erosion using Outcome Measures in Rheumatology Clinical Trials (OMERACT) RA MRI scoring (RAMRIS) [20] and for cartilage loss using the Cartilage Loss Scale (CARLOS) [21]. Missing data were imputed using last observation carried forward (LOCF) or linear extrapolation. Results were compared between the treatment groups using analysis of covariance (ANCOVA) adjusted for baseline scores. Post hoc sensitivity analyses were performed using alternative methods for the imputation of missing data. These alternative methods excluded data from patients who terminated the study early and used baseline scores to impute post-baseline scores based on their similarity to other randomised patients with complete data. The sensitivity analyses were expected to have less discriminatory power than the primary analyses.

For patients who had MRI data (*n* = 183 for measures of joint inflammation; *n* = 142 for measures of joint damage), significant reductions from baseline to week 12 in measures of joint inflammation (synovitis, osteitis and combined inflammation scores) were observed for baricitinib 4 mg compared with placebo (Supplementary Fig. 1). Some measures of joint damage at week 12 (cartilage loss and total joint damage) were also significantly reduced with baricitinib 4 mg compared with placebo (bone erosion was not significantly reduced at this time) (Supplementary Fig. 2). Week 24 scores (*n* = 69) remained stable or were further reduced for bone erosion and total joint damage, but the change in cartilage loss with baricitinib 4 mg at this time was not significantly different versus placebo. The post hoc sensitivity analyses confirmed the findings for bone erosion but not for cartilage loss and total joint damage, for which no significant effects were observed. The beneficial effects of baricitinib 2 mg on the joints were less pronounced than for baricitinib 4 mg, with significant change in measures of combined joint inflammation versus placebo only at week 24, but significant improvements versus placebo at weeks 12 and 24 in bone erosion and at week 12 in total joint damage (Supplementary Figs. 1 and 2).

## Assessment of radiographic progression in phase 3 studies

Radiographic progression following treatment with baricitinib has been evaluated in a number of phase 3 clinical studies, including RA-BEGIN (NCT01711359; mainly [> 91%] DMARD-naïve patients with early RA) [22], RA-BEAM (NCT01710358; inadequate responders to methotrexate with established RA) [10], RA-BUILD (NCT01721057; inadequate responders or those intolerant to csDMARDs with established RA) [9] and the ongoing long-term extension study RA-BEYOND (NCT01885078) [12]. Results obtained with baricitinib doses not approved by the European Medicines Agency are excluded from this review.

In the phase 3 studies, radiographic progression was measured using the van der Heijde modified total Sharp score (mTSS), which includes a score for the extent of joint erosion in 44 joints and the extent of joint space narrowing in 42 joints of the hands and feet [23, 24]. The total score ranges from 0 to 448, with higher scores indicating greater joint damage. Radiographs of the hands and feet were obtained at the screening visit (baseline) and at the endpoint or time of rescue for patients who received rescue treatment: baricitinib 4 mg once daily from week 16 onwards (week 24 in RA-BEGIN) if tender and swollen joint counts had improved by < 20% from baseline at weeks 14 and 16, at the investigator’s discretion. Radiographs were also obtained at the time of study discontinuation if > 12 weeks had passed since the last radiograph. All radiographs were scored centrally and independently by two readers blinded to the chronologic order of the radiographs, patient identity and treatment. The mean score from the two readers was used unless there was disagreement beyond a predefined level, in which case a third reader adjudicated; if the adjudicator provided a score, the two scores closest to each other were used.

Analyses were performed on the modified intent-to-treat populations, consisting of patients with a radiographic assessment at baseline and at least one assessment during the long-term extension study. Missing data, and data missing due to discontinuation or the initiation of rescue therapy, were imputed using linear extrapolation, LOCF or a mixed model for repeated measures. Radiographic progression was defined as a change from baseline to endpoint exceeding 0 or 0.5 Sharp units or the smallest detectable change (SDC) in mTSS, which is the smallest amount of change in score that can be assessed beyond measurement error [25]. Least squares (LS) mean change from baseline in mTSS, erosion score and joint space narrowing score and the proportion of patients with no radiographic progression were compared between treatment groups using ANCOVA, a graphical method for multiple testing or a logistic regression model.

## Baricitinib or baricitinib plus methotrexate versus methotrexate in DMARD-naïve patients (RA-BEGIN)

RA-BEGIN was a phase 3, randomised, double-blind, double-dummy, active comparator-controlled, 52-week study in 588 patients with early RA, limited (up to three weekly doses of methotrexate) or no prior exposure to csDMARDs and no prior exposure to biologic DMARDs (bDMARDs). Patients received methotrexate monotherapy once weekly, baricitinib monotherapy 4 mg once daily or combined treatment. The study is described in detail elsewhere [22]. Patient baseline characteristics are summarised in Table 1. High-sensitivity C-reactive protein (hsCRP) levels were around seven times the upper limit of normal (ULN), and the majority of patients were seropositive (95–97% rheumatoid factor [RF] positive, 89–92% anti-citrullinated protein antibody [ACPA] positive and 87–92% double positive across treatment groups). Baricitinib alone or in combination with methotrexate was superior to methotrexate monotherapy with respect to the proportion of patients with a ≥ 20% response according to American College of Rheumatology criteria (ACR20) at week 24, which was 77% for baricitinib monotherapy (*p* ≤ 0.01 vs methotrexate), 78% for combined therapy (*p* ≤ 0.001 vs methotrexate) and 62% for methotrexate monotherapy.

**Table 1:** Baseline characteristics of patients participating in RA-BEGIN [22], RA-BEAM [10] and RA-BUILD [9]

Characteristic	RA-BEGIN (csDMARD- and bDMARD-naïve)			RA-BEAM (MTX-inadequate responders and bDMARD-naïve)			RA-BUILD (csDMARD-inadequate responders and bDMARD-naïve)		
	MTX once weekly (***N*** = 210)	Baricitinib 4 mg QD (***N*** = 159)	Baricitinib 4 mg + MTX (***N*** = 215)	Placebo (***N*** = 488)	Baricitinib 4 mg QD + background MTX (***N*** = 487)	Adalimumab 40 mg Q2W + background MTX (***N*** = 330)	Placebo (***N*** = 228)	Baricitinib 2 mg QD (***N*** = 229)	Baricitinib 4 mg QD (***N*** = 227)
Age, years	51 ± 13	51 ± 13	49 ± 14	53 ± 2	54 ± 2	53 ± 12	51 ± 13	52 ± 12	52 ± 12
Female, *n* (%)	148 (70)	121 (76)	156 (73)	382 (78)	375 (77)	251 (76)	189 (83)	184 (80)	187 (82)
Duration of RA, years	1 ± 4	2 ± 5	1 ± 3	10 ± 9	10 ± 9	10 ± 9	7 ± 8	8 ± 8	8 ± 8
≥ 1 erosion, *n* (%)	138 (66)	105 (66)	137 (64)	371 (76) ^a^	371 (76)^a^	245 (75)^a,b^	170 (75)	163 (71)	169 (75)
ACPA positive, *n* (%)^c^	193 (92)	142 (89)	192 (89)	424 (87)	427 (88)	295 (89)	172 (75)	169 (74)	163 (72)
RF positive, *n* (%)^d^	203 (97)	155 (97)	204 (95)	451 (92)	439 (90)	301 (91)	171 (75)	177 (77)	173 (76)
ACPA and RF positive, *n* (%)	192 (92)	139 (87)	186 (87)	415 (85)	407 (84)	280 (85)	157 (69)	161 (70)	152 (67)
hsCRP level, mg/L^e^	22 ± 22	24 ± 26	24 ± 29	20 ± 21	22 ± 23	22 ± 21	18 ± 20	18 ± 22	14 ± 15
mTSS units	12 ± 22	13 ± 27	11 ± 20	45 ± 50	43 ± 50	44 ± 51	19 ± 31	26 ± 40	24 ± 40
Erosion score	8 ± 13	9 ± 16	8 ± 12	27 ± 29	25 ± 28	26 ± 29	12 ± 19	16 ± 24	15 ± 23
Joint space narrowing score	4 ± 10	5 ± 12	4 ± 10	18 ± 23	17 ± 23	18 ± 24	7 ± 14	10 ± 18	9 ± 18

Data are reported as mean ± standard deviation unless otherwise indicated

^a^≥ 3 erosions

^b^245 out of 327 patients

^c^ACPA positivity > 10 units/mL (ULN)

^d^RF positivity > 14 units/mL (ULN)

^e^ULN 3 mg/L

*ACPA* anti-citrullinated protein antibody, *bDMARD* biologic disease-modifying antirheumatic drug, *csDMARD* conventional synthetic disease-modifying antirheumatic drug, *hsCRP* high-sensitivity C-reactive protein, *mTSS* van der Heijde modified Total Sharp Score, *MTX* methotrexate, *QD* once daily, *Q2W* every 2 weeks, *RA* rheumatoid arthritis, *RF* rheumatoid factor, *ULN* upper limit of normal

**Fig. 1:**
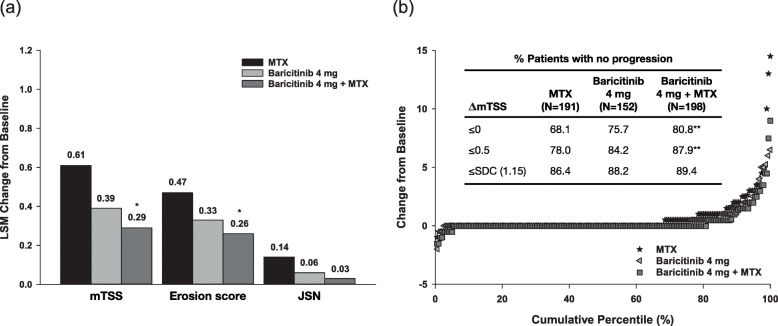
Inhibition of radiographic progression at week 24 with baricitinib, methotrexate and their combination in DMARD-naïve patients with early RA participating in RA-BEGIN [22]. **a** Least squares mean change from baseline in mTSS and its components, and **b** cumulative probability of distribution of change from baseline in mTSS (using linear extrapolation). The table shows the proportion of patients with no radiographic progression, measured as change in mTSS ≤ 0, ≤ 0.5 and ≤ SDC. *p* values for continuous and categorical data were obtained using analysis of covariance and logistic regression, respectively. *p ≤ 0.05, **p ≤ 0.01, ***p ≤ 0.001 versus methotrexate. Δ, change from baseline; JSN, joint space narrowing; LSM, least squares mean; MTX, methotrexate; SDC, smallest detectable change; mTSS, modified total Sharp score. Reproduced with permission from Fleischmann et al. [22]

**Fig. 2:**
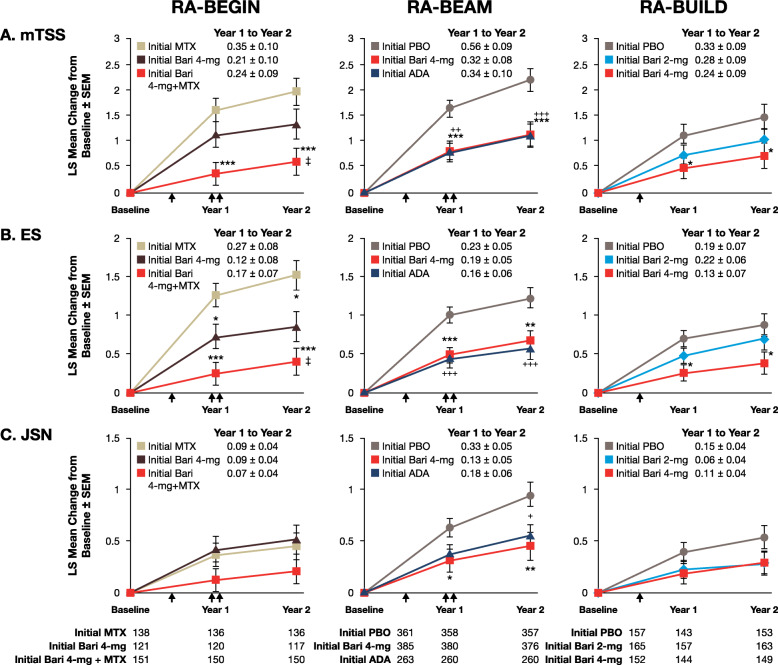
Inhibition of radiographic progression by baricitinib at 1 and 2 years in patients originally participating in RA-BEGIN, RA-BEAM and RA-BUILD, and then RA-BEYOND [12]. Graphs show least squares mean change from baseline (± SEM) in joint damage evaluated using **a** mTSS, **b** erosion score (ES) and **c** joint space narrowing (JSN) score. The tables show the number of patients with available data at each timepoint. Missing data were imputed using linear extrapolation. *p* values were obtained using a mixed model for repeated measures. *p ≤ 0.05, **p ≤ 0.01, ***p ≤ 0.001 for baricitinib 4 mg versus placebo (RA-BEAM, RA-BUILD) or methotrexate (RA-BEGIN); ^+^*p* ≤ 0.05, ^++^*p* ≤ 0.01, ^+++^*p* ≤ 0.001 for adalimumab versus placebo (RA-BEAM); ^‡^*p* ≤ 0.05 for baricitinib 4 mg versus baricitinib 4 mg plus methotrexate (RA-BEGIN). ADA, adalimumab; Bari, baricitinib; LS, least squares; mTSS, van der Heijde modified Total Sharp Score; PBO, placebo; SEM, standard error of the mean. Reproduced with permission from van der Heijde et al. [12]

**Fig. 3:**
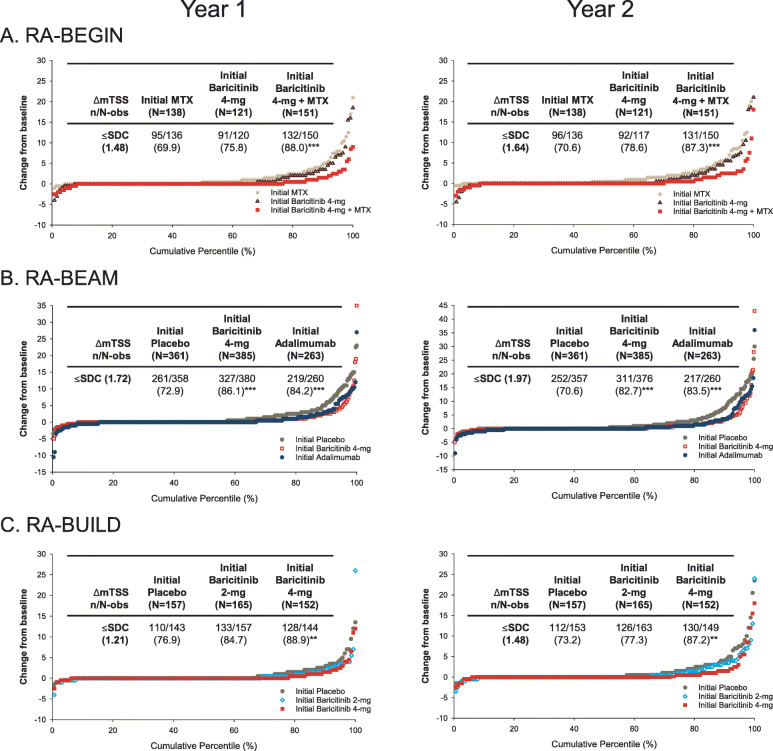
Radiographic progression evaluated using cumulative percentile change in mTSS from baseline at 1 and 2 years by original randomisation for patients participating in **a** RA-BEGIN, **b** RA-BEAM and **c** RA-BUILD, and then RA-BEYOND [12]. Each point represents an individual patient. The table in each figure shows the proportion of patients with no radiographic progression (≤ SDC in mTSS). *p* values were obtained using a logistic regression model with treatment included as a factor. **p ≤ 0.01, ***p ≤ 0.001 for baricitinib 4 mg or adalimumab versus placebo or baricitinib 4 mg plus methotrexate versus methotrexate (RA-BEGIN). Δ, change from baseline; mTSS, van der Heijde modified Total Sharp Score; *n*, number of patients reaching threshold; *N*, number of patients with non-missing baseline and > 1 non-missing post-baseline mTSS values; N-obs, number of patients included in analysis; SDC, smallest detectable change. Reproduced with permission from van der Heijde et al. [12]

At week 24, patients receiving baricitinib (as monotherapy or combined with methotrexate) showed smaller mean changes in mTSS, erosion score and joint space narrowing than patients receiving methotrexate monotherapy. The difference versus methotrexate was statistically significant for mTSS and erosion score for the combination therapy group (Fig. 1a). The proportion of patients who experienced no radiographic progression was also greater with baricitinib than with methotrexate monotherapy, and the difference was statistically significant for the combination therapy group (Fig. 1b). Similar results were observed at week 52, which marked the beginning of the long-term extension study (Figs. 2 and 3a) [12].

**Fig. 4:**
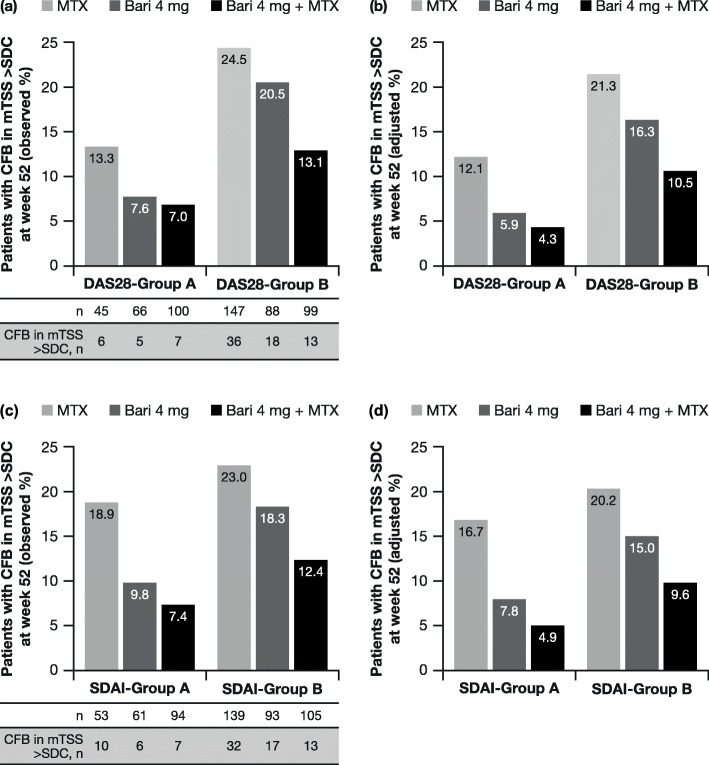
**a** Observed and **b** adjusted proportions of patients with radiographic progression (CFB in mTSS > SDC) at week 52 in patients from RA-BEGIN with (group A) or without (group B) a sustained clinical response, defined as a DAS28-hsCRP score of ≤ 3.2 at weeks 16, 20 and 24. **c** Observed and **d** adjusted proportions of patients with radiographic progression at week 52 in patients with (group A) or without (group B) a sustained clinical response, defined as a SDAI score of ≤ 11 at weeks 16, 20 and 24 [11]. Adjusted proportions were estimated using a multivariable logistic regression model. Bari, baricitinib; CFB, change from baseline; DAS28-hsCRP, Disease Activity Score for 28-joint count based on high-sensitivity C-reactive protein; mTSS, van der Heijde-modified total Sharp score; MTX, methotrexate; SDAI, Simplified Disease Activity Index; SDC, smallest detectable change (1.4 in the RA-BEGIN modified intent-to-treat population). Reproduced with permission from van der Heijde et al. [11]

A post hoc analysis of data from RA-BEGIN evaluated the radiographic progression based on the clinical response to treatment [11]. Patients who achieved a sustained response—defined as a Disease Activity Score for 28-joint count with high-sensitivity C-reactive protein (DAS28-hsCRP) of ≤ 3.2 (*n* = 212) or a Simplified Disease Activity Index (SDAI) score of ≤ 11 (*n* = 209) at weeks 16, 20 and 24—were less likely to show radiographic progression at week 52 than patients who did not achieve a sustained response (*n* = 372) (Fig. 4). For patients who achieved a sustained response, radiographic progression was less likely with baricitinib 4 mg or baricitinib 4 mg plus methotrexate than with methotrexate monotherapy. For patients not achieving a sustained response, radiographic progression was less likely with combination therapy than with either monotherapy.

## Baricitinib versus placebo and an active comparator in inadequate responders to methotrexate (RA-BEAM)

**Fig. 5:**
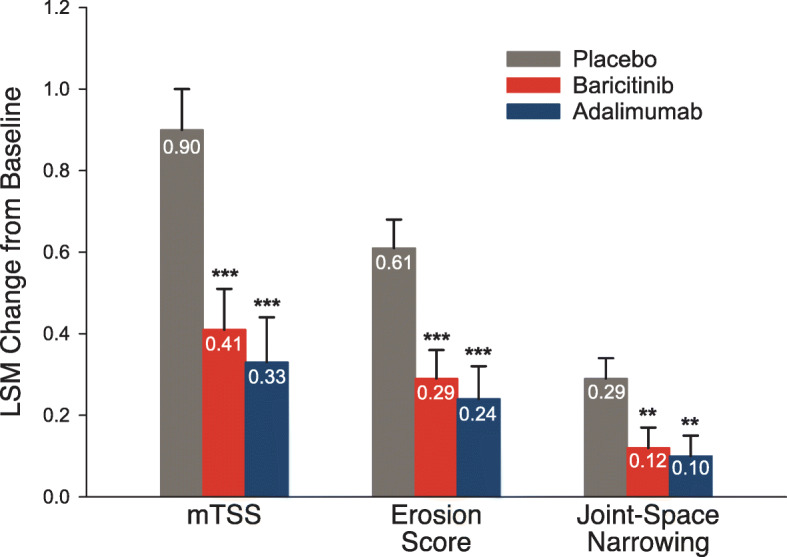
Least squares mean change in radiographic progression from baseline to week 24 in 1234 patients with moderate-to-severe active RA participating in RA-BEAM [10]. Error bars indicate standard error. **p ≤ 0.01, ***p < 0.001 for baricitinib or adalimumab versus placebo. LSM, least squares mean; mTSS, van der Heijde modified Total Sharp Score; RA, rheumatoid arthritis. Reproduced with permission from Taylor et al. [10]

RA-BEAM was a phase 3, randomised, double-blind, placebo- and active-controlled, 52-week study in 1307 patents with moderate-to-severe active RA and an inadequate response to methotrexate. The study design enabled the assessment of changes in structural joint damage in addition to changes in disease activity. Patients received placebo, baricitinib 4 mg once daily or adalimumab 40 mg subcutaneously every other week in addition to existing background therapy. Further details of the study are described elsewhere [10]. Patient baseline characteristics are summarised in Table 1. As in RA-BEGIN, the majority of patients were seropositive (90–92% RF positive, 87–89% ACPA positive and 84–85% double positive across treatment groups) and hsCRP levels were around seven times the ULN. At week 12, the ACR20 response was significantly greater with baricitinib than with adalimumab (70% vs 61%, respectively; *p* = 0.01). In addition, baricitinib proved superior to adalimumab at week 12 with respect to improvements in disease activity. For 1234 patients with baseline and post-baseline radiographic data, both baricitinib and adalimumab significantly reduced radiographic progression at week 24 compared with placebo, and the level of reduction was similar for the two agents (Fig. 5). (Note that for radiographic progression data, no statistical comparison between baricitinib and adalimumab was performed.)

At week 52, patients who initially received baricitinib 4 mg showed significantly smaller mean changes in mTSS, erosion score and joint space narrowing than patients who initially received placebo (Fig. 2). Changes in mTSS and erosion score were similar for baricitinib and adalimumab, but the change in joint space narrowing was not significantly different for adalimumab versus placebo at year 1. The proportion of patients who experienced no radiographic progression was also significantly greater with baricitinib than with placebo, and the results were similar for baricitinib and adalimumab (Fig. 3b) [12].

## Baricitinib versus placebo in inadequate responders to conventional synthetic DMARDs (RA-BUILD)

**Fig. 6:**
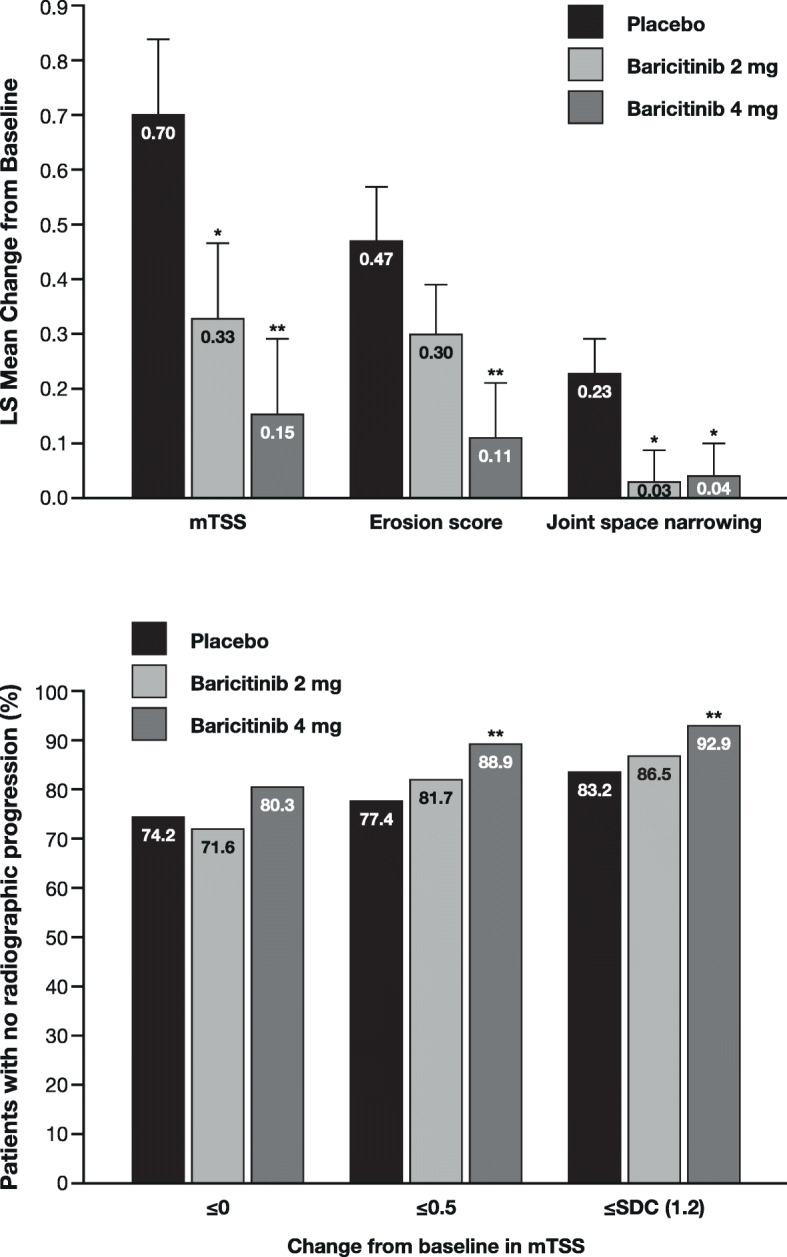
Results of exploratory analyses from RA-BUILD: inhibition of radiographic progression at week 24 for baricitinib versus placebo in 684 patients with moderate-to-severe active RA [9]. **a** Least squares mean change from baseline in mTSS and its components and **b** proportion of patients with no radiographic progression. Error bars indicate standard error. *p ≤ 0.05, **p ≤ 0.01 versus placebo. LS, least squares; mTSS, van der Heijde modified Total Sharp Score; RA, rheumatoid arthritis. Figure 4a reproduced with permission from Dougados et al. [9]

RA-BUILD was a phase 3, randomised, double-blind, placebo-controlled, 24-week study in 684 patients with moderate-to-severe active RA who were naïve to bDMARDs and had shown an inadequate response or intolerance to ≥ 1 csDMARD. Patients received once-daily placebo or baricitinib 2 or 4 mg added to any stable background therapy, including methotrexate. Further study details are presented elsewhere [9]. Patient baseline characteristics are summarised in Table 1. A lower proportion of patients were seropositive (75–77% RF positive, 72–75% ACPA positive and 67–70% double positive across treatment groups) than in RA-BEGIN and RA-BEAM, and hsCRP levels were 5–6 times ULN. The ACR20 response rate at week 12 was significantly greater with baricitinib 4 mg than with placebo (62% vs 39%; *p* ≤ 0.001). Exploratory analyses with respect to radiographic progression revealed a statistically significant reduction in mTSS and joint space narrowing from baseline to week 24 with both baricitinib doses compared with placebo (Fig. 6a). The reduction in joint erosion score versus placebo was significant only for baricitinib 4 mg. The proportion of patients with no radiographic progression was also significantly greater versus placebo for patients receiving baricitinib 4 mg (Fig. 6b).

## Long-term data from DMARD-naïve patients and inadequate responders to csDMARDs, including methotrexate (RA-BEYOND)

Patients who completed baricitinib phase 2 and 3 clinical studies were eligible to enter the ongoing long-term extension study RA-BEYOND. At week 52, patients from RA-BEGIN who received methotrexate or baricitinib 4 mg plus methotrexate were switched to baricitinib 4 mg monotherapy. At the same timepoint, patients from RA-BEAM who received baricitinib 4 mg plus background methotrexate continued to receive the same baricitinib dose plus background methotrexate, while those who received adalimumab on background methotrexate were switched to baricitinib 4 mg plus background methotrexate. At week 24, patients from RA-BUILD who received baricitinib (2 mg or 4 mg) continued to receive the same baricitinib dose, while those receiving placebo switched to baricitinib 4 mg. Radiographic data from patients in RA-BEYOND who participated in RA-BEGIN [22], RA-BEAM [10] or RA-BUILD [9] were analysed at 1 and 2 years or in the event of early study termination [12]. For the RA-BEYOND analyses, baseline was considered to be baseline of the originating study (Supplementary Figs. 3 and 4).

For patients who originally participated in RA-BEGIN [22], those initially receiving baricitinib 4 mg plus methotrexate showed significantly smaller mean changes from baseline in mTSS and erosion score than patients initially receiving methotrexate monotherapy at 2 years (Fig. 2). Patients initially on baricitinib 4 mg monotherapy also showed significantly fewer erosions than patients initially taking methotrexate. The proportion of patients who experienced no radiographic progression was greater with baricitinib plus methotrexate and with baricitinib monotherapy than with methotrexate monotherapy, and the results were statistically significant for baricitinib plus methotrexate versus methotrexate monotherapy (Fig. 3a).

For patients who originally participated in RA-BEAM [10], significantly smaller mean changes in mTSS, erosion score and joint space narrowing were observed at 2 years in patients who initially received baricitinib 4 mg compared with those who initially received placebo (Fig. 2). Changes were similar for baricitinib and adalimumab. At 2 years, the proportion of patients who experienced no radiographic progression was also significantly greater with baricitinib than with placebo, and results were similar for baricitinib and adalimumab (Fig. 3b) [12].

Patients who originally participated in RA-BUILD [9] and received baricitinib 4 mg showed significantly smaller mean changes in mTSS and erosion score at 1 and 2 years than patients who initially received placebo (Fig. 2). Changes with baricitinib 2 mg were smaller than those with placebo, but the differences did not reach statistical significance. Similarly, a greater proportion of patients treated with baricitinib 2 mg or 4 mg experienced no radiographic progression compared with patients who initially received placebo; however, the differences were statistically significant only for the 4 mg dose (Fig. 3c) [12].

For patients originating from RA-BEGIN, those initially receiving methotrexate showed a greater increase in mTSS between years 1 and 2 (0.35 ± 0.10) than those initially receiving baricitinib 4 mg (0.21 ± 0.10) or baricitinib 4 mg plus methotrexate (0.24 ± 0.09). For patients originating from RA-BEAM and RA-BUILD, those receiving placebo showed a greater increase in mTSS between years 1 and 2 (0.56 ± 0.09 in RA-BEAM; 0.33 ± 0.09 in RA-BUILD) than patients receiving baricitinib (0.32 ± 0.08 for baricitinib 4 mg in RA-BEAM; 0.28 ± 0.09 and 0.24 ± 0.09 for baricitinib 2 mg and 4 mg, respectively, in RA-BUILD) (Fig. 2) [12]. Similar results were observed for the erosion score and joint space narrowing, except that the increase in joint space narrowing between years 1 and 2 in RA-BEGIN was similar for patients initially receiving methotrexate or monotherapy with baricitinib 4 mg.

## Radiographic progression according to baseline characteristics

A post hoc analysis of structural damage progression based on clinical response in RA-BEGIN suggested that, independent of treatment (baricitinib 4 mg, methotrexate or a combination), female sex (odds ratio [OR] 2.28, 95% confidence interval [CI] 1.17–4.44; *p* = 0.015), lower body mass index (BMI; OR 0.94, 95% CI 0.89–0.99; *p* = 0.025), smoking (OR 1.92, 95% CI 1.04–3.56; *p* = 0.037), higher hsCRP levels (OR 1.02, 95% CI 1.01–1.03; *p* < 0.001) and higher Clinical Disease Activity Index (CDAI) scores (OR 1.03, 95% CI 1.00–1.05; *p* = 0.038) were significantly associated with an increased probability of such progression [11]. Thus, smokers were at increased risk of structural damage progression compared with non-smokers, while the odds of such progression changed by a factor of 2.28 for being female, 0.94 for each unit increase in baseline BMI, 1.02 for each unit increase in hsCRP levels and 1.03 for each unit increase in CDAI score. The finding of an association between lower BMI and structural damage progression was in line with the results of other studies suggesting that high BMI is associated with a lower risk of such progression, possibly reflecting a phenotype of less aggressive disease in patients with higher BMI [26–29].

Similarly, a post hoc analysis from RA-BEAM showed that lower rates of joint damage progression were observed with baricitinib 4 mg compared with placebo, and the beneficial effect of baricitinib (measured as change in mTSS ≤ 0) was more pronounced in non-smokers than smokers, with 83.7% (304/363) of non-smokers showing a change in mTSS of ≤ 0 compared with 72.9% (78/107) of smokers (interaction *p* value = 0.07) [30]. However, in another post hoc analysis of data from RA-BEAM and RA-BUILD, lower rates of joint damage progression (measured by LS mean change in mTSS from baseline to week 24 using linear extrapolation for missing data) were observed with baricitinib 4 mg compared with placebo irrespective of smoking status (smoker/non-smoker) and body weight (< 60, ≥ 60–< 100 and ≥ 100 kg), with no statistically significant interaction between treatment and smoking status or between treatment and body weight (interaction *p* values 0.942 and 0.566, respectively) [31].

Studies have shown that structural damage progression in patients with RA may be inversely linked to baseline haemoglobin (Hb) levels [32–34], which reflect interleukin-6 and C-reactive protein levels [35, 36]; these, in turn, correlate with structural damage progression [11, 37, 38]. This finding was supported by an analysis of data from RA-BEGIN and RA-BEAM, which showed that lower baseline Hb levels were associated with increased structural damage progression at week 52 (adjusted OR 0.72, *p* = 0.001 in RA-BEGIN; 0.76, *p* < 0.001 in RA-BEAM) [39]. Treatment with baricitinib 4 mg reduced structural damage progression at this time, independent of baseline Hb levels (Supplementary Fig. 5).

## Inhibition of bone loss

In early preclinical studies in a rat model of adjuvant-induced arthritis, treatment with baricitinib 10 mg/kg for 14 days was shown to significantly reduce joint inflammation, ankle width and bone resorption compared with vehicle-treated animals. In addition, baricitinib prevented the joint destruction seen in vehicle-treated animals in the ankles and tarsals as assessed using microcomputed tomography imaging. Similar results were obtained in a mouse model of collagen-induced arthritis [7].

A series of in vivo and in vitro analyses was conducted in parallel with the clinical studies. The results suggested that baricitinib also has an osteoprotective effect, increasing the mineralisation capability of osteoblasts [40]. In a murine model of serum-transfer-induced arthritis, in which mice (*n* = 7–11) received baricitinib 10 mg/kg or vehicle twice daily by oral gavage for 14 days, mean arthritis scores and mean ankle swelling were significantly reduced in baricitinib-treated compared with control mice. While arthritic control mice lost grip strength, trabecular bone volume and thickness and cortical thickness, baricitinib-treated mice showed a significant reduction in inflammation and arthritis-induced bone damage (Supplementary Fig. 6). Similar results were observed with tofacitinib. In vitro studies of murine mesenchymal stem cells that were induced to differentiate into osteoblasts (bone-forming cells) in the presence of baricitinib (30–300 nM) showed that baricitinib increased mineralisation in these cells (Supplementary Fig. 7). Similar studies in human and murine osteoclasts (bone-resorbing cells) showed that baricitinib had no direct impact on osteoclast differentiation or their bone-resorbing capacity. Again, comparable results were observed with tofacitinib [40, 41].

Consistent with the results of preclinical studies, a recent analysis of serum biomarkers in blood samples from 240 patients participating in RA-BUILD showed that treatment with baricitinib 4 mg once daily significantly reduced serum biomarkers of joint synovial inflammation and tissue destruction [42]. At week 4, serum levels of three different biomarkers of synovial inflammation (C1M, C3M and C4M) had decreased by 12–21%, depending on the biomarker (*p* < 0.01), and these reductions were maintained at week 12 (reductions of 11–27%; *p* < 0.001). Decreased serum levels of a biomarker of bone resorption (CTX-I; *p* ≤ 0.05) and a reduction in overall bone turnover of 17% (*p* < 0.01) were also observed with baricitinib 4 mg at week 12. Of note, the decrease in biomarker levels was associated with a decrease in disease activity composite scores, including the SDAI, CDAI, Health Assessment Questionnaire-Disability Index (HAQ-DI) and Disease Activity Score for 28-joint count with erythrocyte sedimentation rate (DAS28-ESR).

## Conclusions

MRI studies have shown that baricitinib 2 mg or 4 mg once daily reduces joint inflammation and damage in patients with moderate-to-severe active RA, although the difference for baricitinib 2 mg versus placebo is not statistically significant. Phase 3 clinical studies have confirmed these findings, showing that, compared with placebo, baricitinib 4 mg significantly inhibits joint inflammation and radiographic joint damage progression in patients with an inadequate response to csDMARDS who are biologic naïve, regardless of csDMARD background medication. The results achieved with baricitinib 4 mg are comparable to those observed with adalimumab. Benefits are also seen with baricitinib 2 mg once daily, but more robust benefit is observed with baricitinib 4 mg. Patient characteristics may influence the effects of baricitinib on radiographic progression. Preclinical studies have shown that baricitinib has an osteoprotective effect, increasing mineralisation in bone-forming cells, but has no direct impact on bone-resorbing cells.

## Supplementary Information

**Additional file 1: Fig. S1.** Least squares mean change from baseline to weeks 12 (left-hand panels) and 24 (right-hand panels) in MRI measures of inflammation: (a) synovitis, (b) osteitis and (c) combined inflammation scores [19]. Error bars indicate standard error of the mean. *p*-values were determined using analysis of covariance. Patient numbers were: placebo, *N* = 48; baricitinib 1 mg, *N* = 27; baricitinib 2 mg, *N* = 29; baricitinib 4 mg, *N* = 26; baricitinib 8 mg, *N* = 24. *p < 0.05, **p ≤ 0.01, ***p ≤ 0.001 versus placebo. LSM, least squares mean; MRI, magnetic resonance imaging. Reproduced with permission from Peterfy et al. [19]

**Additional file 2: **Fig. 2**.** Least squares mean change from baseline to weeks 12 (left-hand panels) and 24 (right-hand panels) in MRI measures of joint damage: (a) bone erosion, (b) cartilage loss and (c) total joint damage [19]. Error bars indicate standard error of the mean. *p*-values were determined using analysis of covariance. Patient numbers were: placebo, *N* = 39; baricitinib 1 mg, *N* = 25; baricitinib 2 mg, *N* = 29; baricitinib 4 mg, *N* = 25; baricitinib 8 mg, *N* = 24. *p < 0.05, **p < 0.01 versus placebo. LSM, least squares mean; MRI, magnetic resonance imaging. Reproduced with permission from Peterfy et al. [19]

**Additional file 3: Fig. S3.** Design of the long-term extension study RA-BEYOND. Reproduced with permission from van der Heijde et al. [12]

**Additional file 4: Fig. S4.** Patient disposition after 2 years of treatment in RA-BEYOND. Reproduced with permission from van der Heijde et al. [12]

**Additional file 5: Fig. S5.** Proportion of patients with RA showing change from baseline in mTSS >SDC at week 52 according to baseline Hb levels in (a) RA-BEGIN and (b) RA-BEAM [39]. ADA, adalimumab; Bari, baricitinib; CFB, change from baseline; Hb, haemoglobin; IR, inadequate response; mTSS, modified Total Sharp Score; MTX, methotrexate; PBO, placebo; SDC, smallest detectable change. Reproduced with permission from Moeller et al. [39]

**Additional file 6: Fig. S6**. Arthritis and bone parameters in mice (*N* = 7–11) with serum-transfer-induced arthritis treated with vehicle (controls), baricitinib 10 mg/kg or tofacitinib 50 mg/kg twice daily for 14 days [40]. The first control group comprised mice without induced arthritis, the second control group mice with induced arthritis. Error bars indicate standard error. *p*-values were determined using one-way analysis of variance (ANOVA). *p < 0.05, **p ≤ 0.01, ***p ≤ 0.001 versus controls. Bari, baricitinib; BV/TV, trabecular bone volume/total volume; Cort, cortical; Ct.Ar/Tt.Ar, cortical bone area/total cross-sectional area inside the periosteal envelope; Ctrl, control; STA, serum-transfer arthritis; Tofa, tofacitinib; Trab, trabecular. Reproduced with permission from Adam et al. [40]

**Additional file 7: Fig. S7.** Increase in mineralised area in murine mesenchymal stem cell-induced osteoblasts at days 5–6 in the presence of baricitinib (30–300 nM) [40]. Error bars indicate standard error. *p*-values were determined using repeated measures ANOVA. **p ≤ 0.01, ***p ≤ 0.001 versus controls. Ctrl, controls. Reproduced with permission from Adam et al. [40]

## Data Availability

The data discussed in this review are not publicly available.

## Abbreviations

ANCOVA: Analysis of covariance
bDMARDs: Biologic disease-modifying antirheumatic drugs
CARLOS: Cartilage Loss Scale
CDAI: Clinical Disease Activity Index
CI: Confidence interval
csDMARDs: Conventional synthetic disease-modifying antirheumatic drugs
DAS28-ESR: Disease Activity Score for 28-joint count with erythrocyte sedimentation rate
DAS28-hsCRP: Disease Activity Score for 28-joint count with hsCRP
DMARDs: Disease-modifying antirheumatic drugs
HAQ-DI: Health Assessment Questionnaire-Disability Index
Hb: Haemoglobin
hsCRP: High-sensitivity C-reactive protein
JAK: Janus kinase
LOCF: Last observation carried forward
LS: Least squares
MRI: Magnetic resonance imaging
mTSS: van der Heijde-modified total Sharp score
OMERACT: Outcome Measures in Rheumatology Clinical Trials
OR: Odds ratio
RA: Rheumatoid arthritis
RAMRIS: OMERACT RA MRI scoring
SDAI: Simplified Disease Activity Index
SDC: Smallest detectable change
STAT: Signal transducers and activators of transcription
ULN: Upper limit of normal
