# Supplementary figures and images for: Baricitinib inhibits structural joint damage progression in patients with rheumatoid arthritis—a comprehensive review
Source: Arthritis Res Ther. 2021 Jan 4;23:3. doi: 10.1186/s13075-020-02379-6 (PMC7784289; doi:10.1186/s13075-020-02379-6)

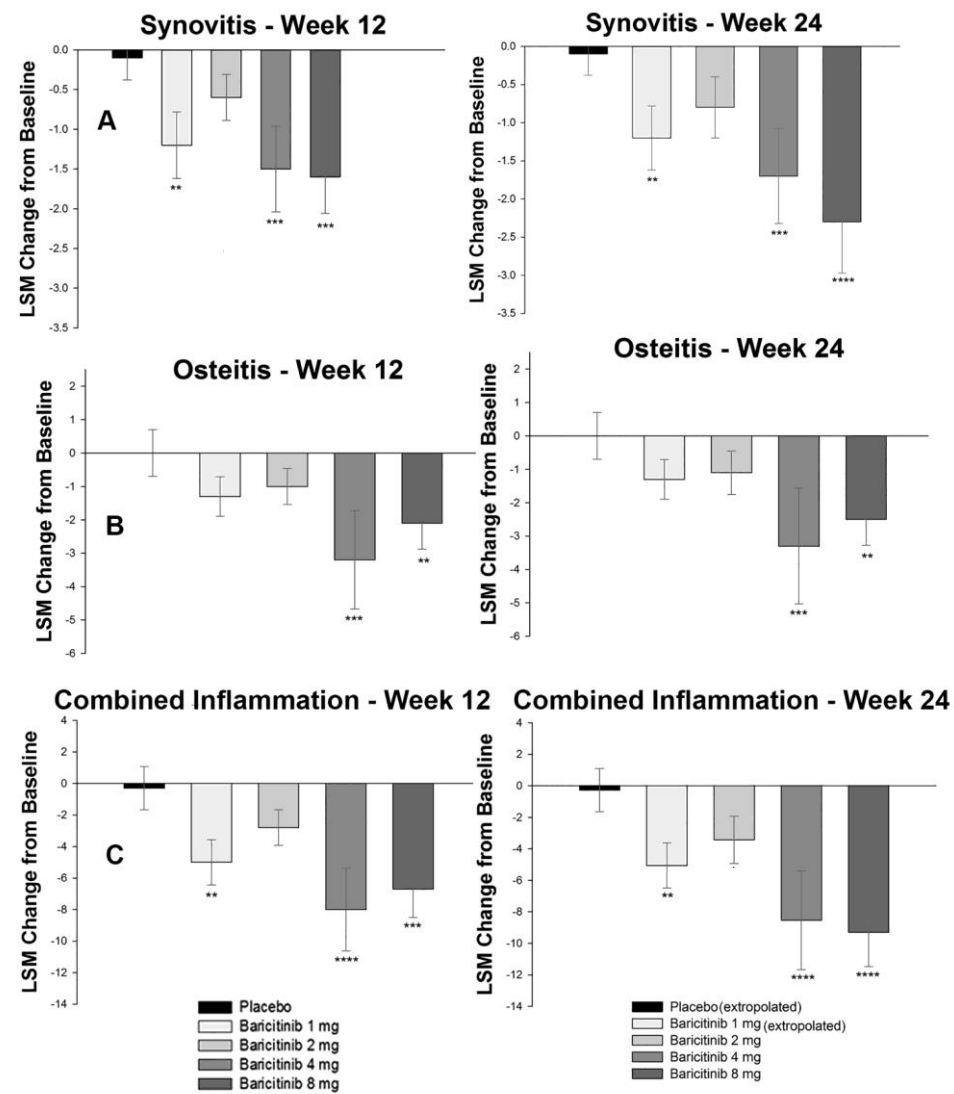

Supplement: Supplementary file 1 — Additional file 1: Fig. S1. Least squares mean change from baseline to weeks 12 (left-hand panels) and 24 (right-hand panels) in MRI measures of inflammation: (a) synovitis, (b) osteitis and (c) combined inflammation scores [19]. Error bars indicate standard error of the mean. p-values were determined using analysis of covariance. Patient numbers were: placebo, N = 48; baricitinib 1 mg, N = 27; baricitinib 2 mg, N = 29; baricitinib 4 mg, N = 26; baricitinib 8 mg, N = 24. *p < 0.05, **p ≤ 0.01, ***p ≤ 0.001 versus placebo. LSM, least squares mean; MRI, magnetic resonance imaging. Reproduced with permission from Peterfy et al. [19] [file 13075_2020_2379_MOESM1_ESM.pdf]

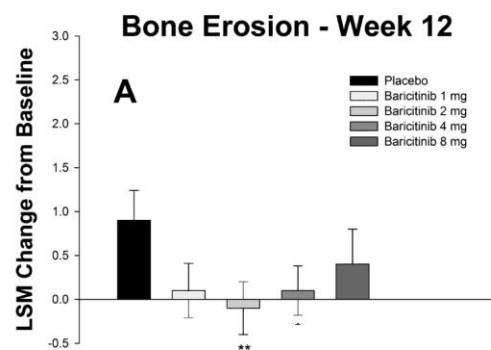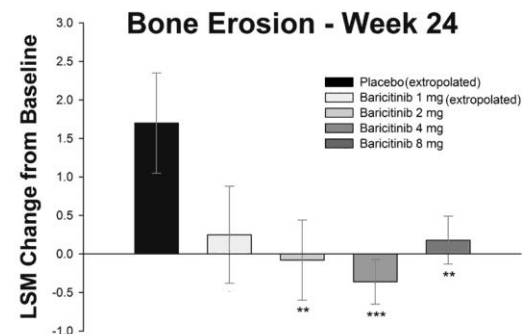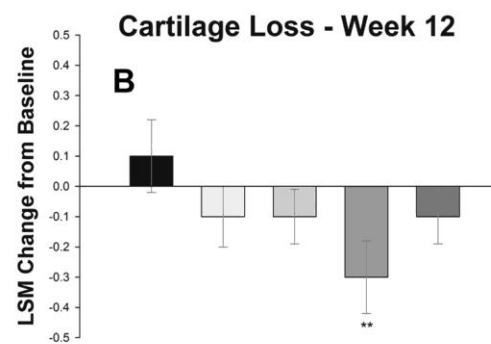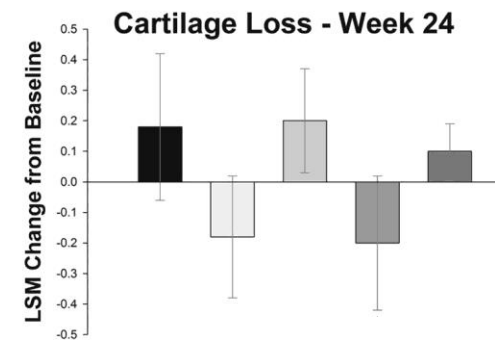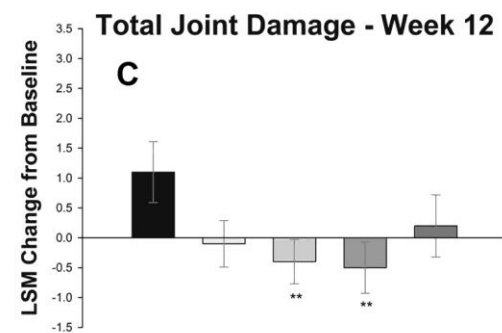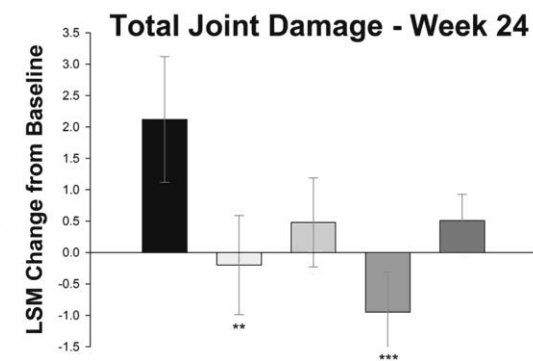

Supplement: Supplementary file 2 — Additional file 2: Fig. 2. Least squares mean change from baseline to weeks 12 (left-hand panels) and 24 (right-hand panels) in MRI measures of joint damage: (a) bone erosion, (b) cartilage loss and (c) total joint damage [19]. Error bars indicate standard error of the mean. p-values were determined using analysis of covariance. Patient numbers were: placebo, N = 39; baricitinib 1 mg, N = 25; baricitinib 2 mg, N = 29; baricitinib 4 mg, N = 25; baricitinib 8 mg, N = 24. *p < 0.05, **p < 0.01 versus placebo. LSM, least squares mean; MRI, magnetic resonance imaging. Reproduced with permission from Peterfy et al. [19] [file 13075_2020_2379_MOESM2_ESM.pdf]

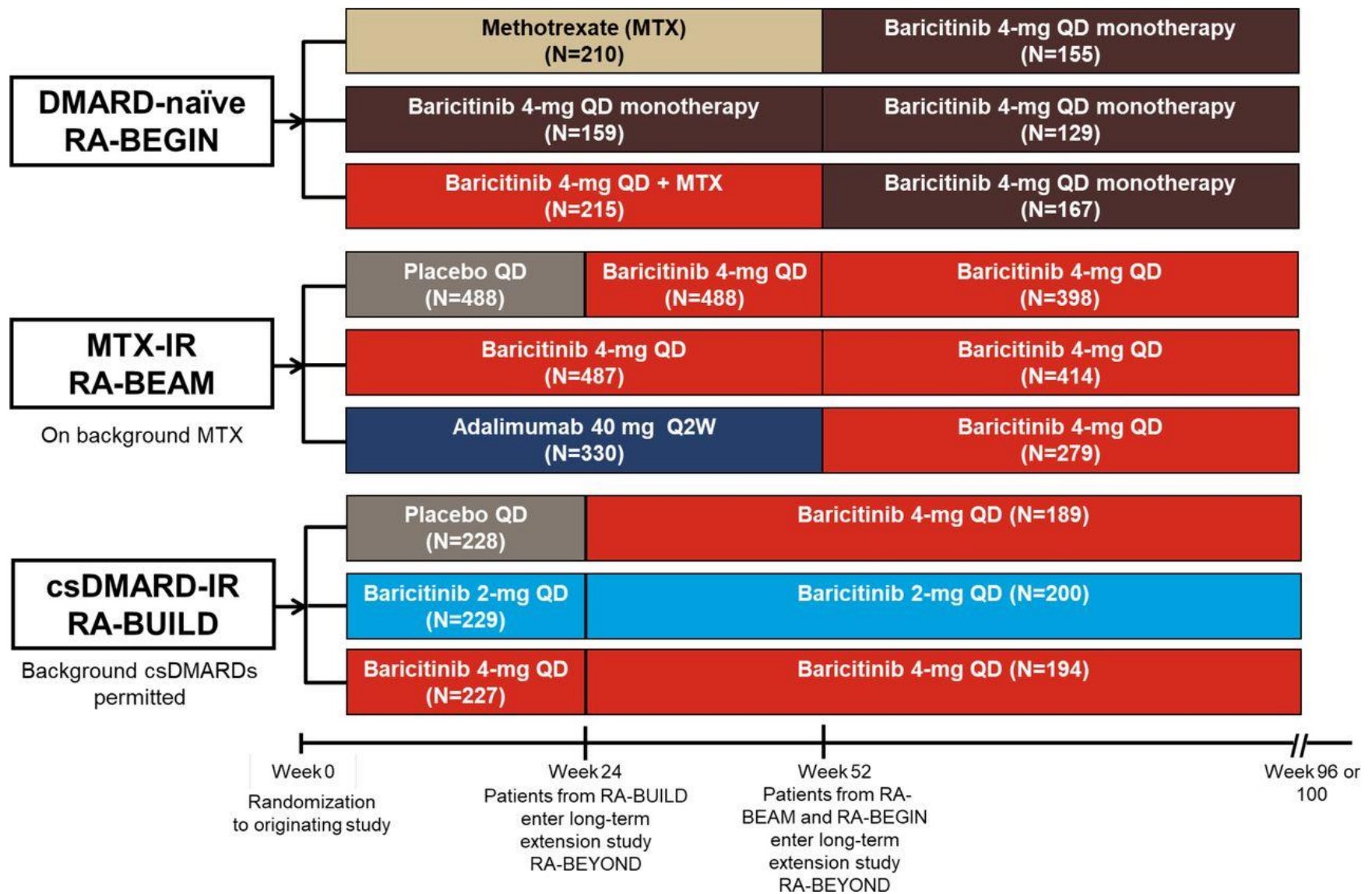

Supplement: Supplementary file 3 — Additional file 3: Fig. S3. Design of the long-term extension study RA-BEYOND. Reproduced with permission from van der Heijde et al. [12] [file 13075_2020_2379_MOESM3_ESM.pdf]

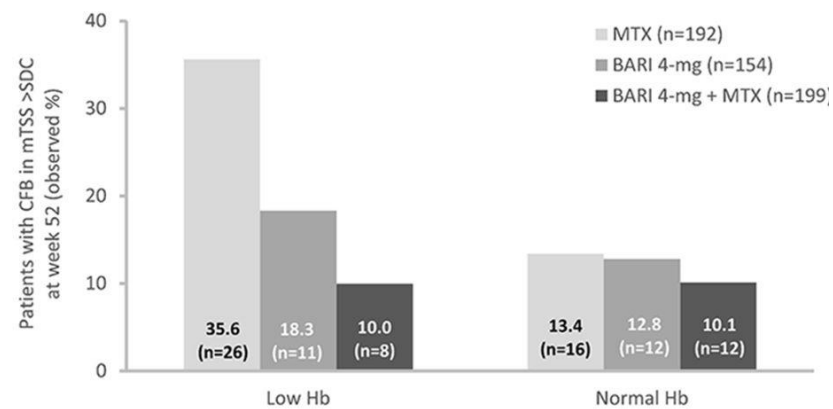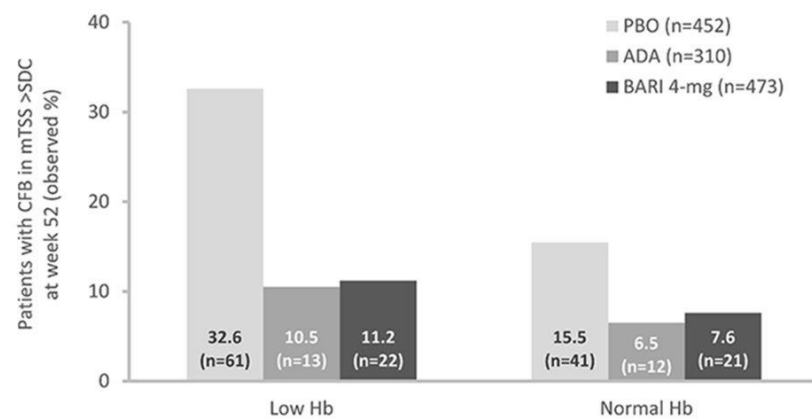

Supplement: Supplementary file 5 — Additional file 5: Fig. S5. Proportion of patients with RA showing change from baseline in mTSS >SDC at week 52 according to baseline Hb levels in (a) RA-BEGIN and (b) RA-BEAM [39]. ADA, adalimumab; Bari, baricitinib; CFB, change from baseline; Hb, haemoglobin; IR, inadequate response; mTSS, modified Total Sharp Score; MTX, methotrexate; PBO, placebo; SDC, smallest detectable change. Reproduced with permission from Moeller et al. [39] [file 13075_2020_2379_MOESM5_ESM.pdf]

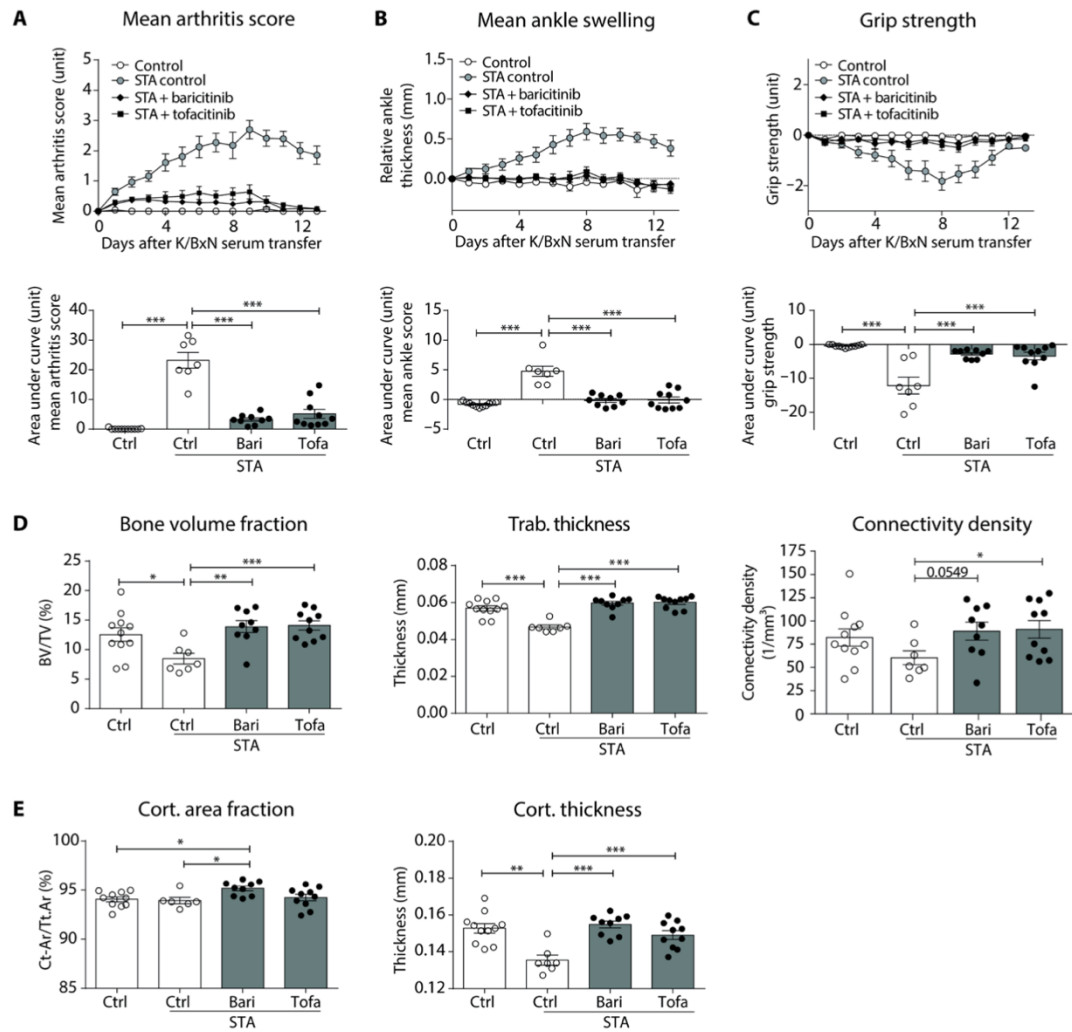

Supplement: Supplementary file 6 — Additional file 6: Fig. S6. Arthritis and bone parameters in mice (N = 7–11) with serum-transfer-induced arthritis treated with vehicle (controls), baricitinib 10 mg/kg or tofacitinib 50 mg/kg twice daily for 14 days [40]. The first control group comprised mice without induced arthritis, the second control group mice with induced arthritis. Error bars indicate standard error. p-values were determined using one-way analysis of variance (ANOVA). *p < 0.05, **p ≤ 0.01, ***p ≤ 0.001 versus controls. Bari, baricitinib; BV/TV, trabecular bone volume/total volume; Cort, cortical; Ct.Ar/Tt.Ar, cortical bone area/total cross-sectional area inside the periosteal envelope; Ctrl, control; STA, serum-transfer arthritis; Tofa, tofacitinib; Trab, trabecular. Reproduced with permission from Adam et al. [40] [file 13075_2020_2379_MOESM6_ESM.pdf]

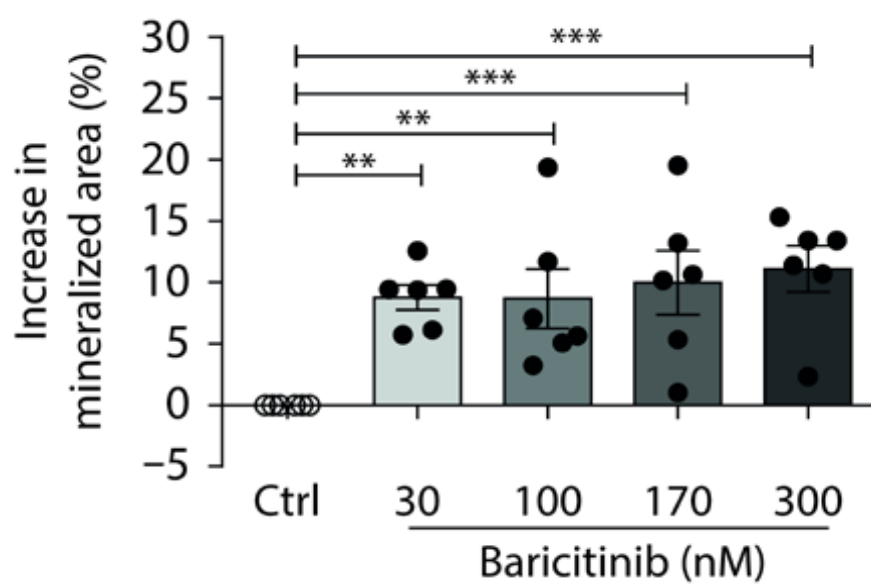

Supplement: Supplementary file 7 — Additional file 7: Fig. S7. Increase in mineralised area in murine mesenchymal stem cell-induced osteoblasts at days 5–6 in the presence of baricitinib (30–300 nM) [40]. Error bars indicate standard error. p-values were determined using repeated measures ANOVA. **p ≤ 0.01, ***p ≤ 0.001 versus controls. Ctrl, controls. Reproduced with permission from Adam et al. [40] [file 13075_2020_2379_MOESM7_ESM.pdf]
