# Supplementary material for: Baricitinib inhibits structural joint damage progression in patients with rheumatoid arthritis—a comprehensive review
Source: Arthritis Res Ther. 2021 Jan 4;23:3. doi: 10.1186/s13075-020-02379-6 (PMC7784289; doi:10.1186/s13075-020-02379-6)

A. Summary of RA-BEGIN after 2 years of treatment

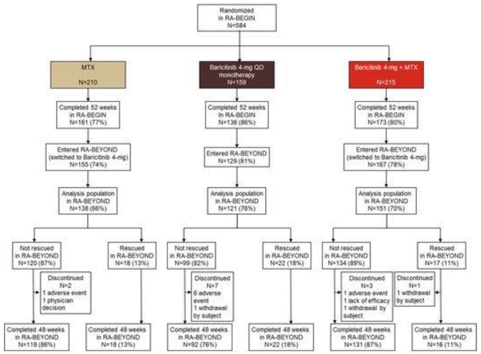

B. Summary of RA-BEAM after 2 years of treatment

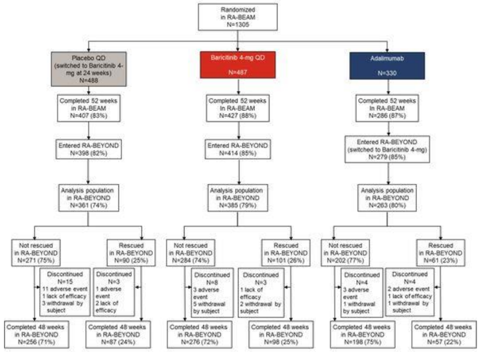

C. Summary of RA-BUILD after 2 years of treatment

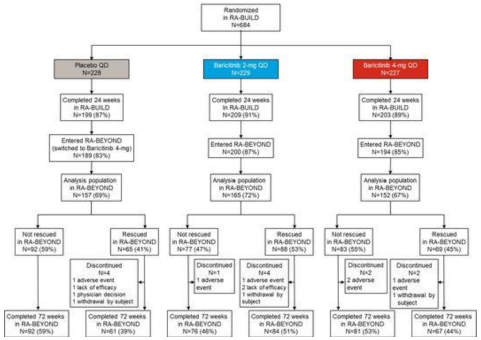

Supplement: Supplementary file 4 — Additional file 4: Fig. S4. Patient disposition after 2 years of treatment in RA-BEYOND. Reproduced with permission from van der Heijde et al. [12] [file 13075_2020_2379_MOESM4_ESM.pdf]
